# Modification of DSSC Based on Polymer Composite Gel Electrolyte with Copper Oxide Nanochain by Shape Effect

**DOI:** 10.3390/polym14163426

**Published:** 2022-08-22

**Authors:** Nur Khuzaimah Farhana, Fatin Saiha Omar, Norshahirah Mohamad Saidi, Goh Zhi Ling, Shahid Bashir, Ramesh Subramaniam, Ramesh Kasi, Javed Iqbal, Swelm Wageh, Hamed Algarni, Abdullah G. Al-Sehemi

**Affiliations:** 1 Centre for Ionics Universiti Malaya, Department of Physics, Faculty of Science, Universiti Malaya, Kuala Lumpur 50603, Malaysia; 2 Department of Applied Physics, Faculty of Science and Technology, Universiti Kebangsaan Malaysia (UKM), Bangi 43600, Selangor, Malaysia; 3 Higher Institution Centre of Excellence (HICoE), UM Power Energy Dedicated Advanced Centre (UMPEDAC), Level 4, Wisma R&D, Universiti Malaya, Jalan Pantai Baharu, Kuala Lumpur 59990, Malaysia; 4 Center of Nanotechnology, King Abdulaziz University, Jeddah 21589, Saudi Arabia; 5 Department of Physics, Faculty of Science, King Abdulaziz University, Jeddah 21589, Saudi Arabia; 6 K. A. CARE Energy Research and Innovation Center, King Abdulaziz University, Jeddah 21589, Saudi Arabia; 7 Research Center for Advanced Materials Science (RCAMS), King Khalid University, Abha 61413, Saudi Arabia; 8 Department of Physics, Faculty of Science, King Khalid University, Abha 61413, Saudi Arabia; 9 Department of Chemistry, College of Science, King Khalid University, Abha 61413, Saudi Arabia

**Keywords:** calcination temperature, copper oxide, DSSC, polymer composite electrolyte, ion diffusion

## Abstract

Solvent evaporation and leakage of liquid electrolytes that restrict the practicality of dye-sensitized solar cells (DSSCs) motivate the quest for the development of stable and ionic conductive electrolyte. Gel polymer electrolyte (GPE) fits the criteria, but it still suffers from low efficiency due to insufficient segmental motion within the electrolytes. Therefore, incorporating metal oxide nanofiller is one of the approaches to enhance the performance of electrolytes due to the presence of cross-linking centers that can be coordinated with the polymer segments. In this research, polymer composite gel electrolytes (PCGEs) employing poly (vinyl butyral-co-vinyl alcohol-co-vinyl acetate) (P(VB-co-VA-co-VAc)) terpolymer as host polymer, tetrapropylammonium iodide (TPAI) as dopant salt, and copper oxide (CuO) nanoparticles as the nanofillers were produced. The CuO nanofillers were synthesized by sonochemical method and subsequently calcined at different temperatures (i.e., 200, 350, and 500 °C), denoted as CuO-200, CuO-350, and CuO-500, respectively. All CuO nanoparticles have different shapes and sizes that are connected in a chain which impact the amorphous phase and the roughness of the surface, proven by the structural and the morphological analyses. It was found that the PCGE consisting of CuO-350 exhibited the highest ionic conductivity of 2.54 mS cm^−1^ and apparent diffusion coefficient of triiodide of 1.537 × 10^−4^ cm^2^ s^−1^. The enhancement in the electrochemical performance of the PCGEs is correlated with the change in shape (rod to sphere) and size of CuO particles which disrupted the structural order of the polymer chain, facilitating the redox couple transportation. Additionally, a DSSC was fabricated and achieved the highest power conversion efficiency of 7.05% with J_SC_ of 22.1 mA cm^−2^, V_OC_ of 0.61 V, and FF of 52.4%.

## 1. Introduction

The effect of climate change is getting serious and is expected to increase the frequency of extreme weather events, such as heatwaves, droughts, etc., in the future. The International Energy Agency (IEA) [1] declared that global warming can be kept under 1.5 °C if net zero emission can be achieved. One of the significant approaches to meet the target is by utilizing solar as the source of energy. Among solar technologies, dye-sensitized solar cells (DSSCs) have garnered attention due to their ability to work efficiently under ambient light illuminations [2,3,4] This feature is vital for photovoltaic systems if electricity is needed under cloudy conditions. In addition, in comparison with other generation solar cells, DSSC fabrication is straightforward and cost-effective since it relies mainly on the use of dye as the photosensitive materials [5,6,7].

Traditionally, a DSSC consists of four major components: (a) a nanocrystalline titanium dioxide (TiO_2_)-based photoanode, (b) a dye as the sensitizer, (c) electrolyte as the medium to ease transfer the ion and regenerate the dye molecules, and (d) a counter electrode that act as catalyst [8,9,10]. Upon illumination on the surface of the DSSC, the dye molecule absorbs the photon and becomes excited then releases an electron. The excited electron is then injected into the mesoporous TiO_2_ layer and transported to the FTO substrate and reaches until the external load. In the electrolyte, $I^{-}$ and $I_{3}^{-}$ ions undergo redox reaction by accepting electrons at counter electrode and releasing the electron to regenerate the oxidized dye and complete the cycle. This complete cycle is repeated billions of times to produce current. In a DSSC, an electrolyte is the most crucial part as it is responsible for ensuring the charge can be transported between positive and negative terminals [10]. It is important for the redox mediator to have good electron transfer kinetics; for instance, (i) an effective redox mediator must have slow electron transfer kinetic at the semiconductor layer to avoid photoexcited electrons being transported to the oxidized element and (ii) fast electron transfer kinetics at the counter electrode to lessen the voltage loss [11]. Over the years, the $I^{-}$/$I_{3}^{-}$ electrolyte has appeared to perform better in the DSSC. The intricate regeneration process for most group 17 and pseudo halogen redox couples (i.e., Br^−^/Br_3_−, SCN^−^/(SCN)_3_−) [12,13] has thus been indicated to limit the DSSC performance. The use of kinetically fast one-electron outer sphere transition-metal complexes (such as Co^2+^/Co^3+^) [14] has caused in low performance DSSC in terms of voltage and current because of increased recombination electrons between the TiO_2_ conduction band and the oxidized redox species. Therefore, in this work, we attempt to improve the transportation of $I^{-}$/$I_{3}^{-}$ to produce better DSSC performance.

The highest efficiency of DSSCs utilizing liquid electrolytes-based $I^{-}$/$I_{3}^{-}$ (LEs) that has been reported is ~14% [15]. However, LEs bring many practical limitations to DSSCs such as leakage issues, degradation of organic compounds, dye desorption, thermal volatility, and corrosion of electrodes [16]. In this viewpoint, various findings have been devoted to replacing LEs, including poly ionic liquid electrolytes (PILs) [17,18], solid polymer electrolytes (SPEs) [19], and quasi-state/gel polymer electrolytes (GPEs) [20]. However, among these options, GPEs seem to be most practical as they will not encounter leaking problems while offering reasonable efficiency (the highest efficiency reported so far is ~9.61%) [21].

A homopolymer has been widely used in polymer electrolytes-based DSSCs to function as a matrix or framework to gel, solidify, and hold a liquid electrolyte which usually contains salt and solvent [22]. Homopolymers such as poly(vinylidene fluoride) (PVDF) [23,24], poly(ethylene oxide) (PEO) [25,26], poly(methyl methacrylate) (PMMA) [27,28,29], poly(acrylonitrile) (PAN) [30], and others are frequently used in the fabrication of GPEs. Unfortunately, the homopolymer’s present qualities in terms of ionic conductivity, electrochemical stability, and tensile strength are insufficient to meet the demands of electrochemical devices. Furthermore, excessive polymer concentration slows ion transport and may reduce the overall ionic conductivity of electrolytes and DSSC photovoltaic performance [31,32,33]. In our previous work, we prepared terpolymer, i.e., poly(vinyl butyral-co-vinyl alcohol-co-vinyl acetate) (P(VB-co-VA-co-VAc) with tetrapropylammonium iodide (TPAI) salt-based GPE and achieved the highest efficiency of 4.62% [34]. This terpolymer was chosen due to the presence of the oxygen atom of vinyl butyl and O-H group of vinyl alcohol, which can interact with TPA^+^ ion from the TPAI salt through Lewis acid–base interaction. This interaction can effectively enhance the transportation of redox pairs in the electrolyte, thereby improving the performance of the device. However, because of the low efficiency obtained, we took another step to boost the efficiency of this polymer system, i.e., by incorporating the polymer with metal oxide nanofillers. 

Generally, metal oxide nanofillers are considered a strong acidity particle and contain cross-linking centers which can form a coordination bond with the host polymer and salts [35,36]. They can increase the amorphous phase and the mechanical strength of polymers owing to their large surface area. Several works which used metal oxide as the nanofiller for DSSC have been reported by Yang et al. (modified nickel oxide (NiO)-based agarose electrolytes with an efficiency of 2.94%) [37], Sethupathy et al. (iron oxide (Fe_2_O_3_)-based blending PVDF-PAN electrolytes with an efficiency of 4.9%) [38], and recently, Saidi et al. (cobalt oxide (Co_3_O_4_) nanocuboids modified PAN-P(PVP-co-VAc) blending electrolyte with an efficiency of 6.46%) [39]. However, the increase in the efficiency by incorporation of metal oxide nanofiller was achieved by varying the concentration or amount of nanofiller, not by the physical structure of nanofiller. In this work, our focus is to investigate the influence of different sizes and different shapes (from sphere to rod) of copper oxide (CuO) particles which are connected into chains on the conductivity of the polymer system. Polymer electrolytes modified with CuO nanofillers are still scarcely reported, especially the ones with unique physical structure.

CuO nanofillers were synthesized using a sonochemical method with a subsequent calcination treatment. In order to investigate the effect of structure and particle size of CuO on the physical and chemical properties of polymer, calcination temperatures were varied (i.e., 200, 350, and 500 °C) [18]. The synthesized CuO nanofillers were then incorporated into the optimized salt based-GPE to form CuO-based polymer composite gel electrolytes (PCGE) samples. The structural crystallinity, surface morphologies, and electrochemical performance of the CuO-based PCGE were analyzed via X-ray diffraction, Fourier transform infrared spectroscopy, and electrochemical studies (electrochemical impedance spectroscopy (EIS), linear sweep voltammetry (LSV) and photocurrent density-voltage (I–V)) 

## 2. Experimental Section

### 2.1. Materials

Poly(vinyl butyral-co-vinyl alcohol-co-vinyl acetate)(P(VB-co-VA-co-VAc) (Mw: 70,000–100,000 g/mol) and Tetrapropylammonium Iodide salt (TPAI) (dried, 99.9%) were purchased from Sigma Aldrich (Merck KGaA, Darmstadt, Germany). Iodine (I_2_, in beads form, 99.9% metal trace basis), ethanol (EtOH, analytical grade, 99.8%), and nitric acid (HNO_3_) (65% *v/v* aqueous solution) were bought from Friedemann Schmidt, Washington, USA. Sodium hydroxide (NaOH, reagent grade, ≥98%, pellets), copper (II) chloride dihydrate (CuCl_2_.2H_2_O, ACS reagent, ≥ 99%), poly(ethylene glycol *t*-octylphenyl ether) (Triton X-100), chloroplatinic acid (H_2_PtCl_6_), and N719 dye (ditetrabutylammonium cis-bis(isothiocyanato)bis(2.2′-bipyridyl-4,4′-di-carboxylato) ruthenium(II)) were purchased from Sigma Aldrich (USA). Titanium dioxide, (TiO_2_) with two different sizes (P90 = 14 nm, and P25 = 21 nm) was acquired from AEROXIDE (Japan). Deionized water (DIW) was used throughout the nanofiller synthesis process. Fluorine-doped tin oxide (FTO) conducting glass plates (sheet resistance = 8 Ω sq^−1^; Solaronix S.A., Aubonne Switzerland)) were cut into 2 × 2 cm sheets and used as substrates for the fabrication of working electrodes and counter electrodes.

### 2.2. Synthesis of CuO Nanofillers

CuCl_2_·2H_2_O (40 mM) were dissolved into DIW and then stirred vigorously for 30 min. Next, NaOH solution (0.2 M) was added slowly into the CuCl_2_·2H_2_O solution and stirred for a while. Then, the solution was subjected to sonication using an ultrasonication probe for 30 min. The amplitude of sonication remained constant at 70%, 120 W (on pulse: 3 s, off pulse: 2 s). After sonication, the solution was centrifuged, and the resulting precipitate was dried at 60 °C for 24 h. Lastly, the powder was calcined at different temperatures; 200 °C, 350 ℃, and 500 °C for 3 h to obtain copper (II) oxide nanofillers, and labeled as CuO-200, CuO-350, and CuO-500, respectively. The powder synthesized without calcination treatment is denoted as Cu(OH)_2_.

### 2.3. Preparation of Polymer Composite Gel Electrolyte-Based P(VB-co-VA-co-VAc) (PCGE) Samples

For the preparation of PCGE samples, the CuO nanofillers were added into the optimized TPAI-P(VB-co-VA-co-VAc)-based GPE (TPAI-4, the GPE incorporated with TPAI salt) that was developed in our previous reported work [34]. In order to investigate the optimal calcination temperature used during the synthesis of CuO nanofiller on the PCGE samples, the weight ratio of TPAI salt and P(VB-co-VA-co-VAc) remained constant at the ratio of 60:40, and the amount of the nanofillers was fixed at 3 wt. %. The specific ratios and concentrations used in this work were fixed based on our previous reported work, where the samples studied were prepared without the addition of filler. As the variable of this work is different calcination temperatures used during the synthesis of CuO nanofiller, we fixed the amount of nanofiller to 3 wt. %. Based on the literature, 3 wt. % is reported as the optimum concentration of filler used to incorporate into the electrolyte system [40,41,42]. No significant effect on the electrochemical performance can be obtained if the filler concentration used is lower than 3 wt. %, and high particle agglomeration occurs if higher concentration of filler is used which can lead to the restriction of ion mobility. 

The suitable amount of TPAI salt and I_2_ were added into the EtOH and stirred continuously until all the materials were dissolved. Then, the CuO was added into the mixture and then allowed to sonicate using the sonicator bath for 20 min until all the CuO nanofillers were well dispersed. Lastly, P(VB-co-VA-co-VAc) was added into the mixture and stirred continuously until the polymer dissolved. Then, the resulting PCGE samples were left to cool down at room temperature. PCGEs incorporated with Cu(OH)_2_, CuO-200, CuO-350, and CuO-500 are labeled as TCu(OH)_2_, TCuO-200, TCuO-350, and TCuO-500, respectively.

### 2.4. Characterizations of CuO Nanofiller and PCGE Samples

The surface morphology of all samples was viewed using field emission scanning electron microscopy (FESEM; Quanta FEG 450 PHILIPS/FEI, Hillsboro, OR, USA) and high-resolution transmission electron microscopy (HRTEM; JOEL JEM-2100F). Structural crystallinity and phase purity of the samples were confirmed by X-ray diffraction (XRD) using an Empyrean X-ray diffractometer (Malvern Panalytical, Malvern, UK) with Cu K_α_ radiation (λ = 15.4056 nm) between 5 and 80°. The complexation of the samples was examined by transmittance mode of Fourier transform infrared spectroscopy (FTIR Perkin Elmer, FTIR-spectrum 400) between 4000 and 650 cm^−1^ with a resolution of 1 cm^−1^. Ionic conductivity measurement of the samples was studied using electrochemical impedance spectroscopy (EIS) technique (HIOKI 3532-50 LCR Hi-Tester, HIOKI E.E Corporation Nagano, Japan) over a frequency range of 50 Hz–5 MHz. The PCGE samples were sandwiched between two stainless steel blocking electrodes with a constant area and thickness of 2.27 cm^2^ and 0.28 cm, respectively. The temperature dependance of PCGE samples was performed using the same technique but at various temperatures (30 to 100 °C) and the samples were allowed to reach thermal equilibrium, about 5–10 min prior to the measurements. The apparent diffusion coefficient of triiodide ion, $D_{I_{3}^{-}}$ in PCGE samples was obtained by performing steady-state linear sweep voltammetry (LSV) analysis using a potentiostat (PGSTAT-128N; Metrohm Autolab, Utrecht, The Netherlands) at a scan rate of 10 mV s^−1^ and the voltage sweep from −0.7 to 0.7 V. The PCGE samples were sandwiched between two Pt electrodes (area of 0.2 cm^2^ and spacer thickness of 48 µm).

### 2.5. Fabrication of DSSC and Its Characterization

**Figure 1:**
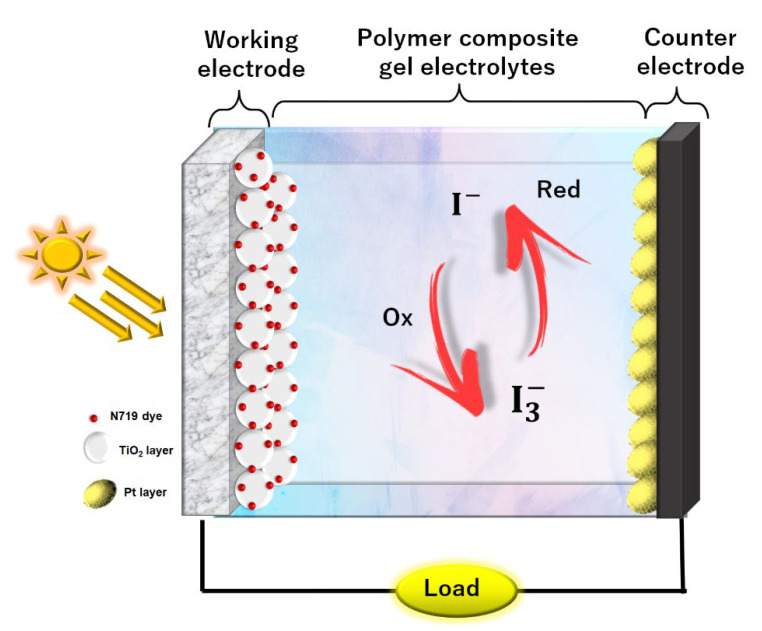
Illustration of DSSC using the configuration of FTO substrate/TiO_2_/N719 ruthenium dye/PCGE/Pt/FTO substrate.

Prior to the preparation of electrodes, the FTO substrate was cleaned using distilled water and EtOH under ultrasonic bath for 5 min. The working electrode was made of two-layer coated TiO_2_, the paste of the first layer TiO_2_ containing TiO_2_ powder, P90, (0.5 g) in 2 mL of Ph = 1 nitric acid. Then, the paste was spin-coated onto the FTO to form a compact layer for better contact between the FTO substrate and TiO_2_, followed by sintering at 450 °C for 30 min. The second layer of TiO_2_ paste consisted of P25 (0.5 g) and three drops of Triton X-100 in 2 mL of pH = 1 of nitric acid which was coated on top of the first layer by using the doctor blade method. Subsequently, the second coat was sintered again at 450 °C for 30 min. Lastly, the coated TiO_2_ working electrode was immersed for 24 h in ethanolic dye solution which consists of 5 mg N719 ruthenium dye, 50 mg chenodeoxycholic acid, and 10 mL of EtOH, while for counter electrode, it was prepared by drop-casting the mixture of 1 g of H_2_PtCl_6_ and 5 mL of EtOH solution on the FTO substrate. The electrode was sintered at 100 °C for 5 min, followed by 450 °C for 30 min. The procedure was repeated twice until the desired Pt electrode was obtained. The DSSC was assembled using the configuration of FTO substrate/TiO_2_/N719 ruthenium dye/PCGE/Pt/FTO substrate as illustrated in Figure 1. 

The assembled cell was then tested under the illumination of 100 mW/cm^2^ simulated sunlight from a Abet Technologies model 10,500 solar simulator with Xenon lamp and processed by computed system Metrohm Autolab potentiostat (PGSTAT128N) for photovoltaic analysis. The active area of DSSC cells was fixed to 0.20 cm^2^.

## 3. Results and Discussion

### 3.1. X-ray Diffraction (XRD)

**Figure 2:**
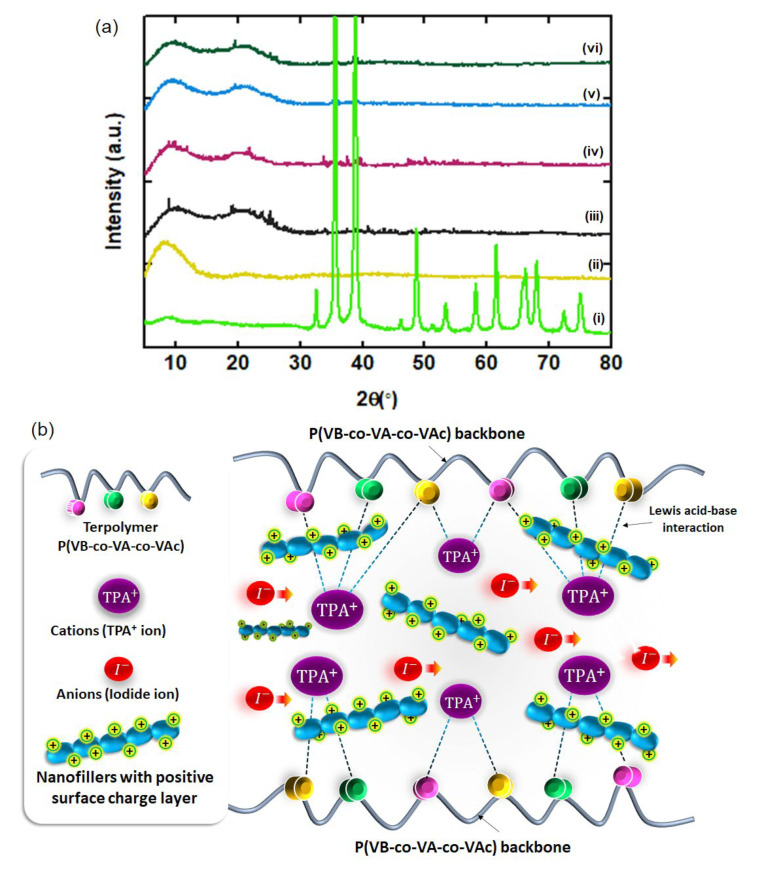
(**a**) XRD pattern for (i) CuO-350 nanofiller, (ii) TPAI-4, (iii) TCu(OH)_2_, (iv) TCuO-200, (v) TCuO-350, and (vi) TCuO-500; and (**b**) illustration of interaction between Lewis acid (TPAI salt and CuO nanofiller) and Lewis base (side chain of P(VB-co-VA-co-VAc) backbone).

The formation of all the nanofillers and the effect of the incorporation of the nanofiller on the crystallinity structure of PCGE samples are shown in Figure 2a and Figure S1, respectively. Cu(OH)_2_ shows a series of sharp peaks, which indicates its crystalline structure, which can be indexed to the orthorhombic phase Cu(OH)_2_ (JCPDS card No. 13-0420) [8]. After calcination, different patterns of the peaks were observed for CuO-200, CuO 350, and CuO-500, which shows that Cu(OH)_2_ was successfully transformed into the monoclinic structure of CuO (JCPDS No. 48-1548) after being calcined at 200 °C (the details of the designation plane for all diffraction peaks is supplied in the Supplementary Materials) [43]. An increase in calcination temperature shows an increase in the intensity of the peaks, indicating the growth of the size of nanofiller. The sonochemical method is widely known to be effective in producing nanomaterials in a relatively short time as compared to other chemical methods such as hydrothermal, sol-gel, or Chemical Vapor Deposition (CVD) [44]. In general, the size of the particle can be manipulated by sonochemical parameters such as power, frequency, and duration [45]. In this work, CuO-350 had the smallest rod size with a diameter of 91 nm. Using the same materials, in order to get nanoparticles with a size smaller than 20 nm, the synthesis process can be conducted either using higher power, higher frequency, or longer duration as these parameters can have a higher impact on the particle size reduction [46,47]. With regard to the relationship between the size of nanoparticles and device efficiency, the smaller the size of nanofiller, the higher the surface area for the Lewis acid–base interaction [48]. A high number of Lewis acid–base interactions between all the elements in the PCGE can lead to the destruction in crystallinity, providing a pathway for the redox mediator mobility and enhancing the device’s efficiency [49,50]. 

TPAI-4 exhibits two peaks: sharp peak at 2θ = 8.5° and broad peak at 2θ = 21.9°, which was reported in our previous work [34]. After the incorporation of the nanofillers, the peak intensity at 8.5° was further reduced and broadened for all the PCGE samples, indicating the decrease in the degree of crystallinity. This decrement was caused by the change of the polymer chain arrangement due to the inclusion of Cu(OH)_2_ and CuO nanofillers in the polymer structure. A couple of new sharp peaks located at 2θ = 35.6° and 38.8° were noticed which correspond to the peak of CuO nanofillers. These peaks were due to the strong Lewis acid of CuO nanofillers which creates a partially positive charge (δ^+^) surface layer to be interacted with the side chain group of P(VB-co-VA-co-VAc) [51]. This interaction is beneficial as it is able to disrupt the rigid structure of the polymer which consequently creates channels for ion mobility ($I^{-}$ and $I_{3}^{-}$) (known as redox mediator in DSSC) (the mechanism of the PCGE samples formation is illustrated in Figure 2b). 

The reduction in the crystallinity of PCGE can be further confirmed by coherent length, τ (Å), which was calculated using Scherrer’s formula as shown in the following equation [52]:

(1) $$\tau \;=\frac{K\lambda }{\beta cos\;\theta }$$

where *K* is shape constant (0.94), *λ* the X-ray wavelength (1.5418 Å), *β* is the FHWM at 2θ in radians, and *θ* is the Bragg angle in radians. The coherent length, τ, for all PCGE samples is tabulated in Table 1. It may be noticed that sample TCuO-350 had the shortest τ among other samples. The shortest τ was correlated to the more amorphous content within the polymer matrix [53]. This suggests that CuO-350 had the largest surface area which led to the highest contact area between CuO and polymers. Therefore, it was concluded that the addition of nanofiller along TPAI reduced the crystallinity of P(VB-co-VA-co-VAc) chains which can aid the enhancement of the mobility of redox couples within the polymer matrix.

### 3.2. Fourier Transform Infrared Studies

**Figure 3:**
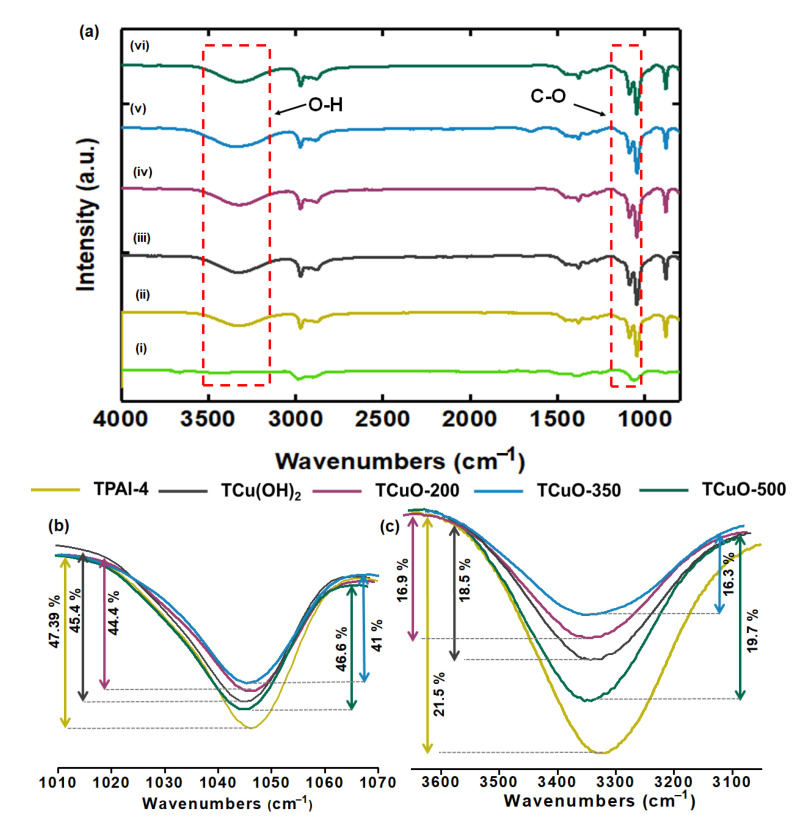
(**a**) FTIR spectra for (i) CuO-350 nanofiller, (ii) TPAI-4, (iii) TCu(OH)_2_, (iv) TCuO-200, (v) TCuO-350, and (vi) TCuO-500, the changes in intensity peak of (**b**) O-H stretching and (**c**) C-O stretching for TPAI-4, TCu(OH)_2_, TCuO-200, TCuO-350, and TCuO-500.

The existence of compounds present in the samples is displayed in Figure 2 and Figure S2. Figure 3a(i) and Figure S2 show that the bands at 3440 and 1628 cm^−1^ for CuO-350, CuO-200, and CuO-500 correspond to the O-H group of water, while the absorption bands observed at 594 cm^−1^ refers to Cu-O stretching modes [54]. Thus, the FTIR results confirm the formation of CuO at all calcination temperatures. Figure S2(i) shows the appearance of stretching modes of O-H groups in Cu(OH)_2_ at the peaks of 3568 cm^−1^ and 3305 cm^−1^. The bands at 1380 and 1060 cm^−1^ indicate the bending mode of the absorbed water in as-prepared of Cu(OH)_2_ powder[55], while at 933 cm^−1^ is attributed to the C-O stretching vibration of metal cation, Cu^2+^ in Cu(OH)_2_. 

The interaction between the nanofillers and functional groups of P(VB-co-VA-co-VAc) is analyzed based on the results shown in Figure 3a(ii–vi). The band of pure P(VB-co-VA-co-VAc) was located at wavenumbers of 3442 cm^−1^ (O-H stretching), 2995 cm^−1^ (C-H), 1131 cm^−1^ (C-O stretching of ether), 1053 cm^−1^ (C-O stretching of alcohol), and 895 cm^−1^ (C-H bending) [34,53]. Upon the addition of TPAI salt, the band of pure P(VB-co-VA-co-VAc) broadened, attributed to the cation coordination with the side chain of P(VB-co-VA-co-VAc) (discussed in our previous work [34]). When salt was dissociated into TPA^+^ and I^-^, TPA^+^ got coordinated with C-O-C group or O-H group, while I^-^ was supposed to be mobile or attached with a methyl group of polymer backbone. When the nanofillers were added (referring to TPAI-4), the band located at 3327 cm^−1^ shifted towards higher wavenumber regions and the band located at 1046 cm^−1^ shifted towards the lower wavenumber regions. Both of the bands are attributed to the band of O-H stretching and C-O stretching, respectively. Furthermore, the band of C-O stretching got broader, and the intensity of the band decreased slightly (can be clearly seen in Figure 3b,c). TCuO-350 had lowest intensity and the broadest band which signifies that it had the highest amorphous content. From the FTIR results, it can be concluded that the TPAI salts dissociated appropriately in the polymer network and the dispersion of the nanofillers modified the polymer chain orderliness which was contributed by the better ion transportations.

### 3.3. Morphology Studies

**Figure 4:**
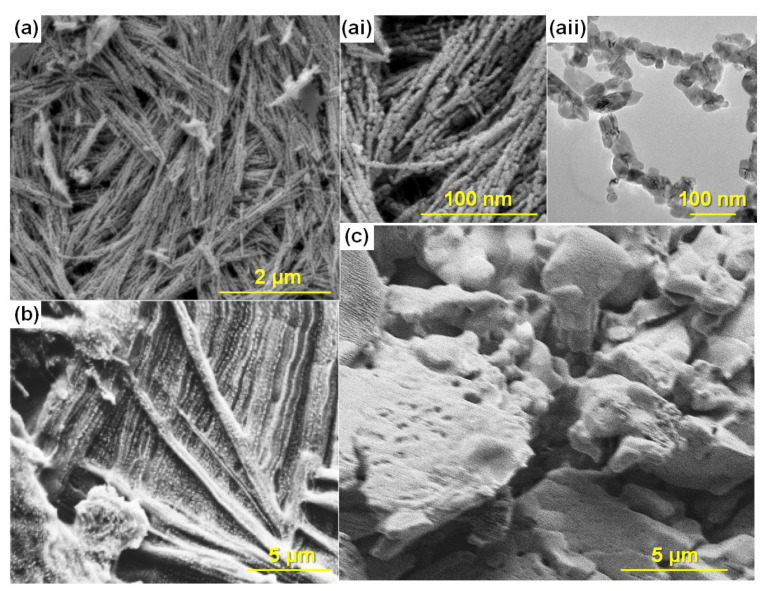
FESEM image of CuO-350 at (**a**) low magnification image, (i) high magnification image, (ii) HRTEM image of CuO-350; FESEM image for the PCGE sample of (**b**) TPAI-4 and (**c**) TCuO-350.

The morphology of CuO-200, CuO-350, and CuO-500 was viewed via FESEM as displayed in Figure 4a and Figure S3. The dimensions of the particle varied significantly with increasing calcination temperature. This indicates that the calcination temperature only led to the growth of the particles. The size and the shape of the nanofillers at different temperatures are presented in Table S1. The morphology of CuO-350 as shown in Figure 4a(i,ii) started to look like a chain (range of length: 1.58 μm) which consists of several nanorods (average length: 227 nm; diameter: 91 nm) when the sample was calcined at 350 °C. The shape turned from rod to sphere at 500 °C (shown in Figure S3(iii)). The HRTEM analysis was conducted for CuO-350 nanofillers (CuO-350 was chosen because of its highest ionic conductivity in PCGE form), and the result is displayed in Figure 4a(ii). It was further confirmed that the particles interconnected to each other and formed a small chain. These structures can be beneficial for the electrochemical reaction in the electrolytes which are able to facilitate the ion diffusion within the electrolyte and create percolation pathways faster rate of ion mobility [56,57]. The smooth and homogenous surface of pure P(VB-co-VA-co-VAc) (refer Figure S3) was modified to a coarser surface after the addition of TPAI salt as shown in Figure 4). After the addition of nanofiller, the PCGE samples in Figure 4c and Figure S3 show a crumpled and rougher surface compared with the TPAI-4, which is most likely due to the interaction between the TPA^+^ nanofiller and/or nanofiller-polymer. The surface morphology of all PCGE samples showed the different roughness because of different particle sizes of the nanofillers. 

### 3.4. Electrochemical Impedance Studies

#### 3.4.1. Ionic Conductivity Studies

**Figure 5:**
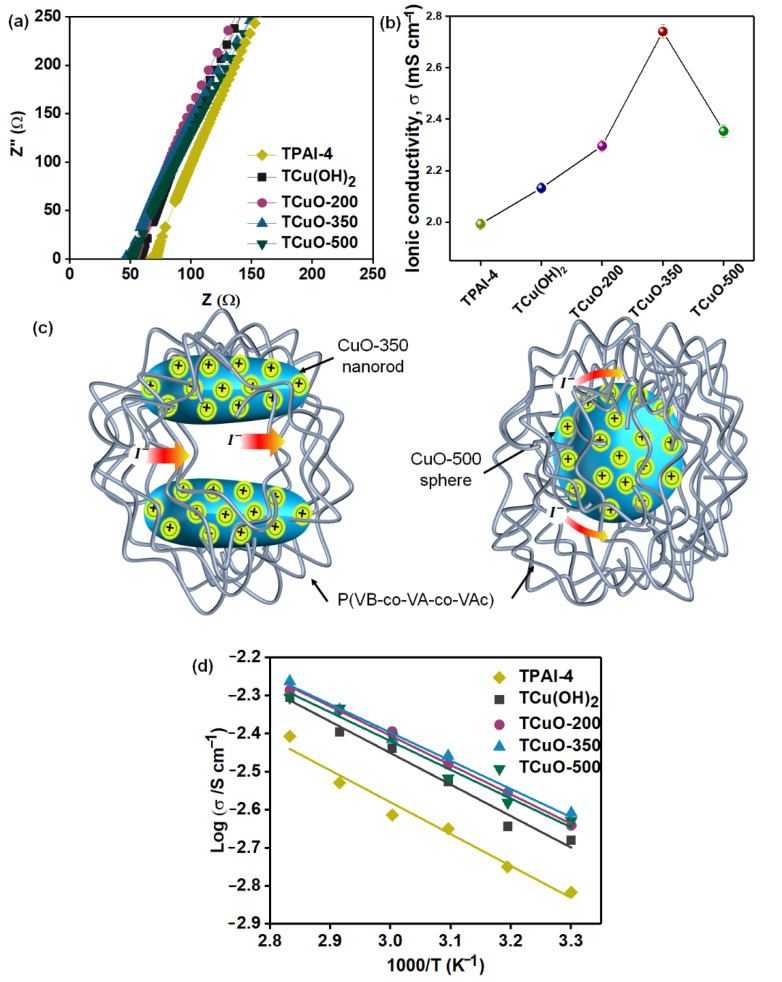
(**a**) Cole–Cole plot of all PCGE samples, (**b**) ionic conductivity of PCGE samples, (**c**) illustration of interaction between different shape of copper oxide nanofiller in the PCGE samples, and (**d**) temperature dependance conductivity plot for all PCGE samples.

The ionic conductivity of PCGE samples was investigated through EIS in a frequency range of 50 Hz–5 MHz. Generally, the impedance plot showed two regions at room temperature: (i) a semicircular at high-frequency region and (ii) a spike at low-frequency region. The semicircular region is described as the bulk conduction process, whereas the spike is ascribed to the growth of free charges at the interface of electrode-electrolytes [58,59]. However, in our PCGE samples (Figure 5a), the semicircular was not observed at high frequency which means that the current mobile charge carriers were mainly from ions. Only anions contribute to the enhancement of ionic conduction, in this case, ions were $I^{-}$ and $I_{3}^{-}$, known as the redox mediator. This is because TPA^+^ is known as the bulky cation which makes it difficult to be mobile within the gel electrolyte as compared to the lighter redox mediator [60]. The disappearance of the semicircle was caused by random dipole alignment of side chains in the polymer networks, resulting in non-capacitance of the electrolyte [61]. Other than that, the intercept of the spike at the real axis determined the estimated value of bulk resistance, R_b_, of the PCGEs sample and is presented in Table 2. The ionic conductivity of the PCGE samples was calculated from the complex impedance spectrum given by Equation (2);

*σ*_*DC*_ = *t*/*R*_*b*_*A* (2)

where *σ* is the *DC* ionic conductivity obtained in S cm^−1^ *t* is the thickness of the PCGE samples in cm, *R_b_* is bulk resistance in Ω, and *A* is the area of the blocking electrode in cm^2^. Based on Figure 5b, TCuO-350 has the highest ionic conductivity (2.74 mS cm^−1^), followed by TCuO-200> TCuO-500>TCu(OH)_2_>TPAI-4. As expressed in Equation (3), the ionic conductivity is dependent on the concentrations of the free ions, ion charge, and the mobility of ions.

*σ* = *neμ* (3)

where *σ* is the ionic conductivity in S cm^−1^, *n* is density of free ions, *e* is the ion charge, and *μ* is the mobility of ions.

The addition of nanofillers into the polymer-salt system (TPAI-4) shows an increment in the value of ionic conductivity as shown in Figure 5b. Upon incorporation of Cu(OH)_2_/CuO nanofiller, the structural disorderliness of P(VB-co-VA-co-VAc) as side chains of polymer then interacts with two possible Lewis acids (in this case; Cu(OH)_2_/CuO nanofiller and TPAI salts). This interaction resulted in: (i) increasing the free volume at the interphase between Cu(OH)_2_/CuO nanofillers and P(VB-co-VA-co-VAc) matrix, thus improving the movement of ions [62,63]; and (ii) the creation of the percolation paths that can accelerate the ionic transportation. The strong acidity of metal oxide nanofillers can make their surface partially positive and causes the redox couple to be preferentially absorbed on the surface of nanofillers, creating percolation pathways [64]. The highest conductivity observed at TCuO-350 was due to its rod shape. TCuO-350 showed a higher conductivity than TCuO-500 which is mainly due to the factor of an aspect ratio of the CuO nanofiller. Generally, nanorod particles have a higher aspect ratio than sphere particles which provide a longer and continuous platform for the activity with the surrounding environment [65,66,67]. This aspect is beneficial for PCGE as the CuO nanorod in TCuO-350 allows a large number of interactions to occur between the partially positive-charged acidic surface groups (Cu-O) and the negatively charged redox couple, which eventually creates longer percolation pathways for ion mobility within the polymer. This phenomenon is not easily achieved by TCuO-500 as its CuO sphere filler has a limited platform for allowing the same interaction due to its sphere shape which has a lower aspect ratio than nanorod [68]. (Figure 4c illustrates the shape effect of interaction between rod and sphere in the PCGE samples). Although, TCuO-500 exhibits lower ionic conductivity than TCuO-350, it still outperformed the TPAI-4 sample (polymer-salt system). This shows that the presence of filler can improve the properties and electrochemical performance of GPE regardless of the shape effect. Similar findings were reported by Do’s group on their work comparing the ionic conductivity of PEO-based solid polymer electrolyte with incorporation of Fe_2_O_3_ rod and sphere forms [69]. The ionic conductivity of PCGE samples was further studied in the temperature range of 30–80 °C and the variation of ionic conductivity against temperature is shown in Figure 4d. It was noticed that ionic conductivity is directly proportional to the temperature for all PCGE samples. The higher the temperature, the higher the segmental motion of the polymer backbone, thus, the higher the ionic conductivity of the PCGE samples [9,70]. The linear variation of Log σ versus 1000/T fitted to the Arrhenius model. The Arrhenius model is expressed as Equation (4) and the activation energy, $E_{a}$ (which is defined as the minimum energy required by ions in order to move) is calculated and tabulated in Table 2 [71].

(4) $$\sigma =\sigma_{0}\;exp^{\frac{-E_{a}}{kT}}$$

where $\sigma_{0}$ is the constant pre-exponential factor, $E_{a}$ is the activation energy (eV), T is absolute temperature (K), and k is the Boltzmann constant (8.617 $\times 10^{-5}$ eV K^−1^). Among the PCGE samples, it was found that TCuO-350 achieved the lowest $E_{a}$, which supports its highest ionic conductivity. 

#### 3.4.2. Dielectric Properties Studies

The purpose of the study of the relative permittivity (ε^*^_r_ (ω)) was to comprehend the polarization effect at the electrode and electrolyte boundary and compare the ionic relaxation time with the ionic conductivity. The relative permittivity is defined as the dimensionless ratio of the permittivity (ε_r_ (ω)) over the permittivity of free space (ε_0_). It is also exhibited as a function of angular frequency containing the real and imaginary parts. 

The dielectric constant is defined as the relative permittivity of a dielectric material. It is a vital parameter to calculate the electrical charges capacity of a dielectric material that can be achieved and collected. Dielectric loss measures the loss of energy that occurs in the structure of a material due to the motion of ions and the alignment of dipoles as the polarity of an energy field changes promptly. The dielectric constant (ε’) and the dielectric loss (ε”) can be determined using the Equations (5) and (6) [72]:

(5) $$\varepsilon^{\prime }=\frac{{\left(Z\prime \prime \right)}^{2}}{\omega C_{0\;}({\left(Z^{\prime }\right)}^{2}+\left(Z\prime \prime {)}^{2}\right)}$$

(6) $$\varepsilon^{\prime \prime }=\frac{{\left(Z^{\prime }\right)}^{2}}{\omega C_{0\;}({\left(Z^{\prime }\right)}^{2}+{\left(Z\prime \prime \right)}^{2}}$$

where C_0_ is the vacuum capacitance, and ω is the angular frequency (2πf).

**Figure 6:**
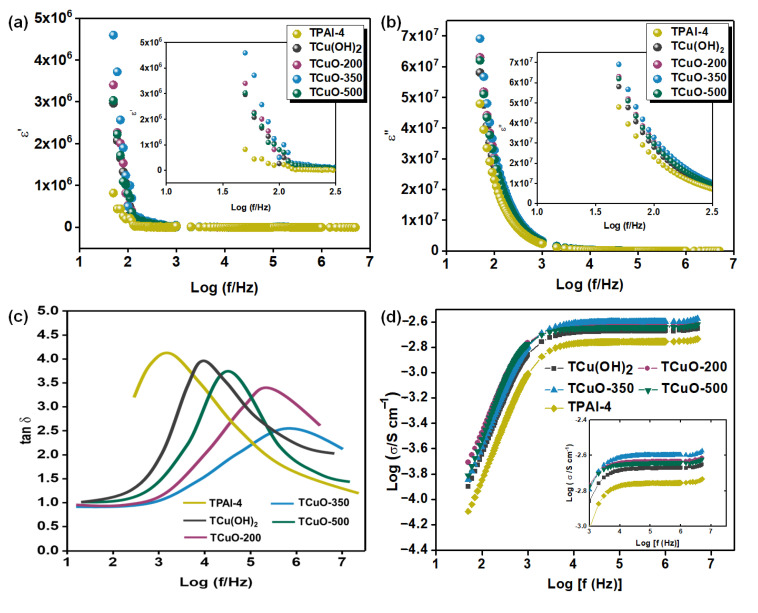
(**a**) Dielectric constants, (**b**) trends of dielectric loss, (**c**) variation of tangent loss, and (**d**) trend of AC conductivity as a function of logarithm frequency for all developed PCGE samples.

From the findings of dielectric behavior investigations, it is possible to better understand ionic transport. The frequency dependence of ε′ for each sample is shown in Figure 6a. The value of ε’ for each sample was extremely high in the low-frequency zone and fell non-linearly as the frequency increased. This is due to the fact that ε’ represents the amount of charge that may be stored within the electrolytes. When applied at low frequency, the free ions inside the polymer matrix may react fast and aggregate at the electrode–electrolyte interface [62]. Only a small amount of charge agglomerated at the blocking electrode contact, resulting in a nominal value of ε’. This is because TCuO-350 has the highest concentration of free ions, which causes this impact. These findings were in line with those found in pervious ionic conductivity investigations.

Figure 6b shows the frequency dependence of ε” of the PCGE based on CuO at different calcination temperature systems. At low frequencies, ε” was enormous, but it approached zero as it rose in frequency. This is a similar pattern to that established in the ε’. In this instance, the material dissipation of electromagnetic energy is called dielectric loss. As a result, Figure 6b indicates that the heat energy dissipated the fastest when a low-frequency external field was given to the PCGE samples. The same justification as in ε’ applies here. The rapidly reversing electric field caused the free ions to oscillate about their equilibrium point. As a result, the ions were incapable of dissipating and transporting heat via collision [73]. Figure 6b shows that TCuO-350 showed highest dielectric loss as an increased number of densities of charge carriers undergo internal friction and create a large amount of heat energy loss. 

#### 3.4.3. Loss Tangent and Frequency Dependance Conductivity Studies

The dipole relaxation for the developed PCGE samples was analyzed by using dielectric relaxation over a broad range of frequency. By using Equation (7), the tan *δ* plot as a function of the logarithm frequency for PCGE samples is displayed in Figure 5.

(7) $$\tan\delta =\frac{\varepsilon^{\prime \prime }}{\varepsilon^{\prime }}$$

The pattern of tan *δ* plot increased with the frequency until reaching an optimal value, and then decreased in the high frequency area. The increment of tan *δ* at the low-frequency area owing to the ohmic element was more dominant over the capacitive element, while the peak of tan *δ* related to the upper limit energy transfer for a specific frequency and indicated better relaxing dipoles in the electrolytes. The decrement at high frequency was assigned to the independent-ohmic element from frequency and the increase of reactive component [74]. With addition of nanofiller, the relaxation peak shifted toward the high-frequency region as shown in Figure 6c. This suggests that a decline in relaxation time and, therefore faster segmental relaxation within the electrolytes [51]. The shifting of peaks was because of the increase in amorphous content and reduced the relaxation time after the addition of the nanofiller. This reduced relaxation time indicates that the ions easily migrate from one vacant site to another [75]. From Figure 6c, it was observed that TCuO-350 had the maximum shift in tan *δ* compared to TPAI-4, representing the lowest relaxation time due to the improved amorphous phase within the PCGE samples. The presence of longer nanorod in TCuO-350 facilitated the mobility of the redox couple and reduced the duration of relaxation. As described in Section 3.4.1, the longer nanorod of CuO-350 nanofiller enhanced the ionic conductivity by improving movement of ions (more amorphous content) and the creation of the percolation paths [68]. Therefore, it reduced the relaxation of time as conductivity increased. The increase in relaxation of time for TCuO-500 was expected due to the shorter conductive path and bigger diameter of sphere for CuO-500 nanofiller that minimized the interaction with polymer chains and reduced the amorphous content in the PCGE samples which led to reducing the mobility of redox couples.

Figure 6d depicts the frequency-dependent conductivity, σ_AC_ for all the PCGE samples. A general pattern in the frequency-dependent conductivity was noticed for all PCGE samples which consisted of two regions: (i) low frequency dispersion region and (ii) plateau region at high frequency [73,76]. At the low frequency region, the conductivity was assigned to the polarization influence at the interface of electrolyte–electrode. When the frequency decreased, there was more charge accumulation at the interface of the electrolyte–electrode resulting in decrease in the conductivity, while at the plateau region, the conductivity was almost found to be frequency independent and dc conductivity, σ_DC_ could be extracted. This behavior is supported by Jonscher’s universal power law, as expressed in Equation (5) [74,77];

σ (ω) = σ_DC_ + A ω^n^ (8)

where σ_DC_ is the DC conductivity (S cm^−1^), A is pre-exponential factor, and n is the fractional exponent between 0 and 1. The σ_AC_ of the prepared PCGE samples was determined by extrapolating the plateau region on the *y*-axis from Figure 6d and tabulated in Table 2. TCuO-350 exhibited the maximum σ_AC_ with value of 2.51 mS cm^−1^. This was very close to the σ_DC_ obtained in a previous EIS study (see Figure 5b). 

### 3.5. Linear Sweep Voltammetry Studies

**Figure 7:**
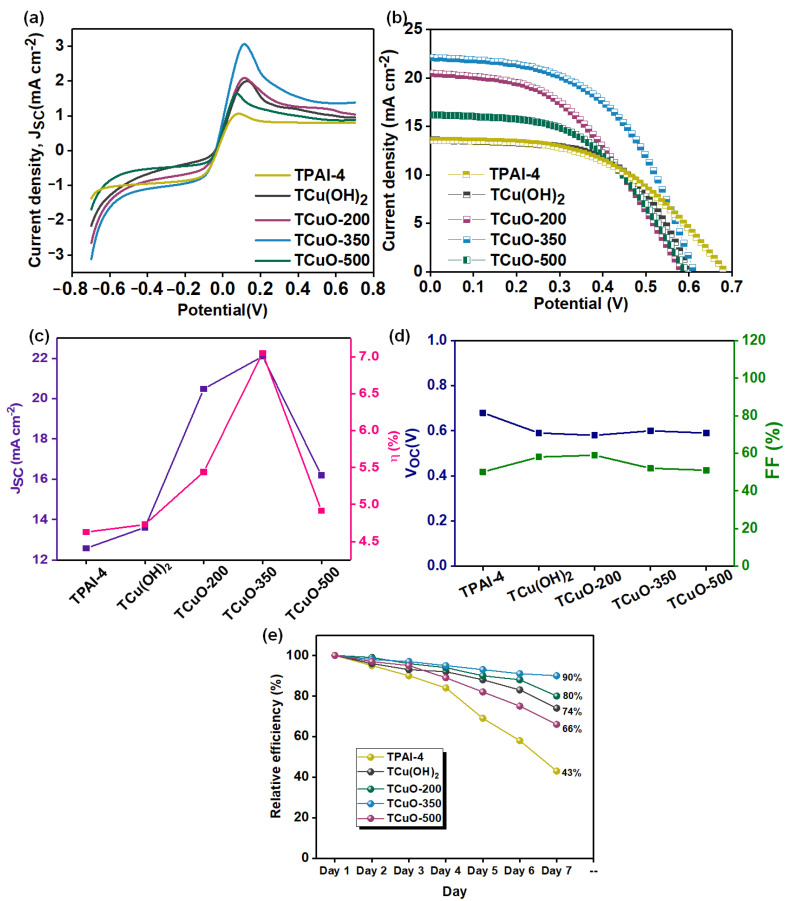
(**a**) LSV curve, (**b**) J-V curve, (**c**) parameter of short-circuit current density (*J_SC_*) and conversion efficiency (*η*) graph, (**d**) parameter of open-circuit voltage (*V_OC_*) and fill factor (*FF*) graph, and (**e**) stability of DSSC for all PCGE samples.

Linear sweep voltammetry (LSV) measurements were conducted to examine the effective diffusion coefficient of triiodide ions ($D_{I_{3}^{-}}$). Figure 7 displays the characteristics of LSV curves for the PCGE samples and the ($D_{I_{3}^{-}}$) values are tabulated in Table 3. The chemical reactions I of the Pt/electrolyte/Pt electrochemical occurs due to the applied potential are as follows [63,78]:

(9) $$I_{3}^{-}+2e\;\to 3I^{-}\;(\mathrm{reduction})$$

(10) $$3I^{-}\;\to \;I_{3}^{-}+2e\;(\mathrm{oxidation})$$

The current densities attained saturation for both polarities at above 0.3 V which indicates the equilibrium steady-state conditions. It was noted that the limiting current indicated to triiodide ions as iodide concentration was larger than the concentrations of iodine. Therefore, limiting current density ($J_{lim}$) could only be used to obtain the apparent diffusion coefficient of triiodide ($D_{I_{3}^{-}}$) in PCGE samples according to following relation (Equation (11)) [9,79]:

(11) $$D_{I_{3}^{-}\;}=J_{lim}\frac{d}{2nFC}$$

where *d* refers to the PCGE samples thickness (24 μm), *n* is the number of electrons (*n* = 2), *F* is Faraday constant, and *C* is the initial concentration of $I_{3}^{-}$ ions which is equivalent to the I_2_ concentration in mol/cc.

In Figure 7a, the peaks were observed at 0–0.2 V due to failure of flux *I*_3_^−^ at the electrode to sustain with the rise in diffusion layer above the electrode [33]. Hence, the limiting current density is determined when the current does not change with potential (V = 0.3–0.7 V). Based on Table 3, the maximum limiting current density was observed in TCuO-350 sample at current density of 2.48 mA cm^−2^ and thus, it further demonstrated the highest improvement in $D_{I_{3}^{-}}$ from 5.94 × 10^−6^ cm^2^ s^−1^ (TPAI-4) to 1.25 × 10^−5^ cm^2^ s^−1^. This increment in the ion diffusion demonstrated by TCuO-350 was due to the higher amorphous phase and longer continuous pathway in the polymer electrolytes after the addition of CuO-350. Other than that, the inclusion of the nanofillers into the polymer electrolyte contributed to the catalytic behavior and improved the cation accumulation by the P(VB-co-VA-co-VAc) chains, causing higher value of photocurrent as discussed in photovoltaic studies.

### 3.6. Photovoltaic Performance Studies

The photocurrent density-voltage (J-V) curve for the developed DSSC containing all the PCGE samples is presented in Figure 7b. The parameters of DSSCs such as short-circuit current density (*J_SC_*), photoconversion efficiency, PCE (*η*), open-circuit voltage (*V_OC_*), and fill factor (*FF*) are illustrated in Figure 7c,d. As can be seen, all the PCGEs-based DSSCs showed an increment in *J_SC_* and *η* after the addition of the nanofillers. The influence of these nanofillers on ionic conductivity was consistent with the $D_{I_{3}^{-}}$. The results denote that the ion transport and ionic conductivity in the PCGE samples were particularly related to the $D_{I_{3}^{-}}$. In Figure 7c, the *J_SC_* value of the cells increased with addition of Cu(OH)_2_, CuO-200, and CuO-350 nanofiller. Then, it decreased with addition of CuO-500 nanofiller. The results indicate that the ionic conductivity and the $D_{I_{3}^{-}}$ were the factors determining the *J_SC_* of the cells. If nanoparticles are used as the filler in polymer composite, the crystallinity of polymer composite will be disrupted as compared to the pure polymer, regardless of the degree of crystallinity of the nanoparticles [52,80]. In this work, the effect of shape and size of the nanoparticles which were manipulated via the calcination temperature was more dominant than crystallinity as these effects influenced the arrangement of polymer chains by the formation of Lewis acid–base interaction between the polymer and the filler. This interaction increased the amorphous volume fraction in the polymer, which in turn enhanced the ion mobility and hence the ionic conductivity [51]. As the faster ion mobility occurs within the polymer electrolytes, it will improve the dye regeneration kinetics and enhance the photocurrent as well as the efficiency of the device.

For better interaction between the components within the electrolytes, a strong acidic metal oxide is preferable compared to base and neutral metal oxide type as stated by Croce et.al [81]. As we incorporated copper oxide (one of strong acid metal oxide) [82] in the PCGEs, it was proven that it is able to improve the segmental mobility for redox couple transportation due to this interaction and hence, enhance the performance of the cells. Among all PCGE samples, TCuO-350 exhibited the highest *η* i.e., 7.05%. The optimum nanorods size which is favorable for the redox couple movement in the electrolytes and high triiodide diffusivity (as we discussed in pervious section) elevated the dye molecules’ regeneration [83]. Hence, a greater number of electrons could be transported to the TiO_2_ semiconductor surface to complete the cycle. As a result, TCuO-350 achieved the highest *J_SC_* and *η*, among others. Table 4 reports the efficiency of reported work on DSSC work for the comparison in recent literature. It was noticed that the *η* in this work that employed CuO as nanofiller were higher and comparable to the other transition metal oxide nanofiller.

On the other hand, in Figure 7d, the value of *V_OC_* (which is related to the potential difference between the TiO_2_ fermi level and redox couple level) is slightly dropped. It has been reported that the adsorption and accumulation of free cations in the electrolytes onto mesoporous TiO_2_ led the TiO_2_ Fermi level to move closer to the valence band. In TPAI-4, TPA^+^ ions were mostly coordinated to the side chain of P(VB-co-VA-co-VAc), thus giving a potential difference of 0.65 V because of fewer free TPA^+^ ions to alter the position of Fermi level. However, with the addition of CuO nanofiller, more TPA^+^ free ions were adsorbed and accumulated on TiO_2_ mesoporous working electrodes as polar groups of P(VB-co-VA-co-VAc) tended to coordinate with CuO nanofiller. This caused the Fermi level of TiO_2_ to be moved towards the valence band and gave minor changes in *V_OC_* value [89,90]. In addition, the *FF* value was observed to be slightly changed after the addition of nanofillers which could be related to the charge accumulation at the interface of working electrode/PCGE samples due to irregular movements of charge, rate transfers of electron at interface[91], dye regeneration, electron recombination[92], and the stickiness between PCGE samples, working electrode, and counter electrode [64,65,66].

Figure 7e demonstrates the long-term stability of DSSC over a week (once per day) for all PCGE samples. It can be noticed that *η* steadily declined from Day 1 to Day 7 which was due to the evaporation of the EtOH and improper sealing of the DSSC device. The TPAI-4 had lower stability with *η* remaining around 43% after the 7th day, whereas after the addition of nanofiller, the stability of the device improved and TCuO-350 obviously showed the highest stability, where it retained its *η* by 90% after a week. In comparison, the *η* of TCu(OH)_2_, TCuO-200, and TCuO-500 after the 7th day remained at 74%, 80%, and 66%. The high stability demonstrated by TCuO-350 proved that the longer nanorod of CuO nanofiller (CuO-350) provided percolation paths for better redox couple diffusion and led to the higher stability performance than the sphere nanofiller.

## 4. Conclusions

CuO nanofillers with different particle sizes (Cu(OH)_2,_ CuO-200, CuO-350, and CuO-500) were synthesized using a sonochemical method, followed by calcination at various temperatures. The crystallinity and structure of the nanofillers and the polymer systems were confirmed by XRD and FTIR analyses. It was noted that the calcination temperature was an essential factor, which could influence the structure, growth, and electrochemical performance of the nanofiller. With increasing calcination temperature, the particle size increased, and the structure changed from an interconnected chain of small rod to sphere. Among all the nanofillers, CuO-350 exhibited the highest dispersibility and large effective surface area that could facilitate a better redox couple transportation within the PCGE samples. TCuO-350 exhibited the highest ionic conductivity (2.54 mS cm^−1^) and maximum apparent diffusion coefficient of triiodide of 1.537 × 10^−4^ cm^2^ s^−1^ as compared to the other PCGE samples. This was due to the disorderliness of the polymer network by the interaction of Lewis acid–base between CuO nanofiller, TPAI salt, and the side chain of P(VB-co-VA-co-VAc). In summary, the augmentation of ionic conductivity may be due to the following influences: (i) presence of the interconnected chain of small rod shape effect, (ii) formation of continuous conducting pathways, (iii) increase in amorphous domain, and (iv) improved redox couple mobility. Moreover, the highest surface area of CuO-350 also provided a continuous conducting path for better redox couple diffusion. In addition, the device DSSC employing TCuO-350 achieved the highest power conversion efficiency (7.05%) with *J_SC_* of 22.1 mA cm^−2^, *V_OC_* of 0.61 V, and *FF* of 52.4%. 

## Figures and Tables

**Table 1:** τ of all the PCGE samples.

Samples	Peak 1			Peak 2		
	FHWM (⁰)	2θ (⁰)	τ (Å)	FHWM (⁰)	2θ (⁰)	τ (Å)
TPAI-4	4.61	8.9	4.61	6.4	21.9	2.28
TCu(OH)_2_	5.76	10.1	2.42	11.8	20.3	1.91
TCuO-200	5.10	9.9	2.73	12.2	19.7	1.15
TCuO-350	6.18	10.5	2.25	16.0	19.5	0.88
TCuO-500	5.66	10.6	2.49	12.5	19.6	1.12

**Table 2:** The bulk resistance (R_b_), activation energy (E_a_), and direct-current conductivity (σ_dc_) values for all PCGE samples.

PCGE Samples	R_b_ (Ω)	E_a_ (eV)	$\sigma_{\mathrm{AC}}\;(\times$10^−3^ S cm^−1^)
TPAI-4	64.5	0.166	1.58
TCu(OH)_2_	58.4	0.164	1.99
TCuO-200	54.3	0.153	2.14
TCuO-350	45.5	0.147	2.51
TCuO-500	52.9	0.150	2.24

**Table 3:** The value of limiting current density and apparent diffusion coefficient of triiodide for all PCGE samples.

PCGE Samples	J_lim_ (mA cm^−1^)	$D_{I_{3}^{-}}$ (×10^−6^ cm^2^ s^−1^)
TPAI-4	1.22	5.94
TCu(OH)_2_	1.34	6.74
TCuO-200	1.93	9.70
TCuO-350	2.48	12.5
TCuO-500	1.49	7.49

**Table 4:** Comparison of reported efficiency for transition metal oxide as nanofiller based PCGE used in the DSSC and TCuO-350.

Electrolytes	Type of Nanofiller	η (%)	Reference
**P(VB-co-VA-co-VAc)/TPAI/CuO-350 (TCuO-350)**	**CuO-350 (Nanorod)**	**7.05**	**In the present work**
PVDF-HFP: PMMA/BMII/LiI	TiO_2_ (nanocrystalline)	7.08	[84]
Agarose/LiI/NMP	NiO	2.02	[85]
HPC/KI	Co_3_O_4_ (nanocuboid)	5.80	[86]
PEO/NaI	ZnO	6.94	[87]
PEG/LiI/MPII	Modified-ZrO_2_	5.60	[88]

## Data Availability

The data presented in this study are available on request from the corresponding author.

## Supplementary Materials

The following supporting information can be downloaded at: https://www.mdpi.com/article/10.3390/polym14163426/s1, Figure S1: XRD pattern for (i) Cu(OH)2 nanofiller, (ii) CuO-200 nanofiller and (iii) CuO-500 nanofiller., Figure S2: FTIR spectra for (i) Cu(OH)_2_ nanofiller, (ii) CuO-200 nanofiller and (iii) CuO-500 nanofiller and Figure S3: FESEM micrographs for (i) Cu(OH)_2_ nanofiller, (ii) CuO-200 nanofiller, (iii) CuO-500 nanofiller, (iv) Pure P(VB-co-VA-co-VAc), (v) TCu(OH)_2_, (vi) TCuO-200 and (vii) TCuO-500.; Table S1: Size and shape of Cu(OH) and CuO nanofiller at different calcination temperature.
