# Supplementary material for: Modification of DSSC Based on Polymer Composite Gel Electrolyte with Copper Oxide Nanochain by Shape Effect
Source: Polymers (Basel). 2022 Aug 22;14(16):3426. doi: 10.3390/polym14163426 (PMC9413727; doi:10.3390/polym14163426)
Supplement: Supplementary file 1 [file polymers-14-03426-s001.zip › polymers-1797299-supplementary.pdf]

Supplementary files

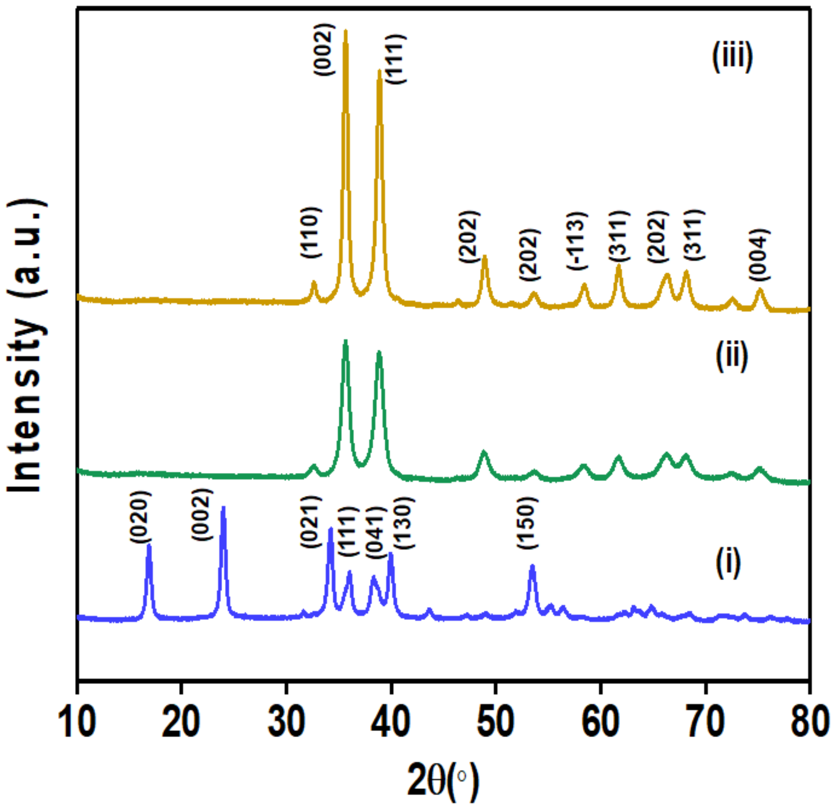

Figure S1: XRD pattern for (i) $Cu(OH)_2$ nanofiller, (ii) CuO-200 nanofiller and (iii) CuO-500 nanofiller.

The effect of calcination temperature on the structure of the samples is studied via XRD diffractograms as shown in Figure 1 and Figure S1. $Cu(OH)_2$ shows a series of sharp peaks which indicates its crystalline structure at 16.8°, 23.9°, 34.2° , 35.9°, 38.3°, 40.5°, and 53.3°. These peaks can be indexed to (020),(021), (002),(111), (041), (130) and (150) planes of orthorhombic phase $Cu(OH)_2$ (JCPDS card No. 13-0420), respectively [22]. After calcination, different patterns were observed for CuO-200, CuO 350 and CuO-500 which signifies that $Cu(OH)_2$ was successfully transformed into the CuO. The peaks of CuO are located at 32.6°, 35.6°, 38.8°, 48.8°, 58.3°, 61.6°, 66.3°, 68.1°, 72.4°, and 75.1° in 2θ, corresponding to (110), (002),(111), (202), (202), (-113), (311), (202), (311) and (004) planes of monoclinic CuO (JCPDS No. 48-1548), respectively [23].

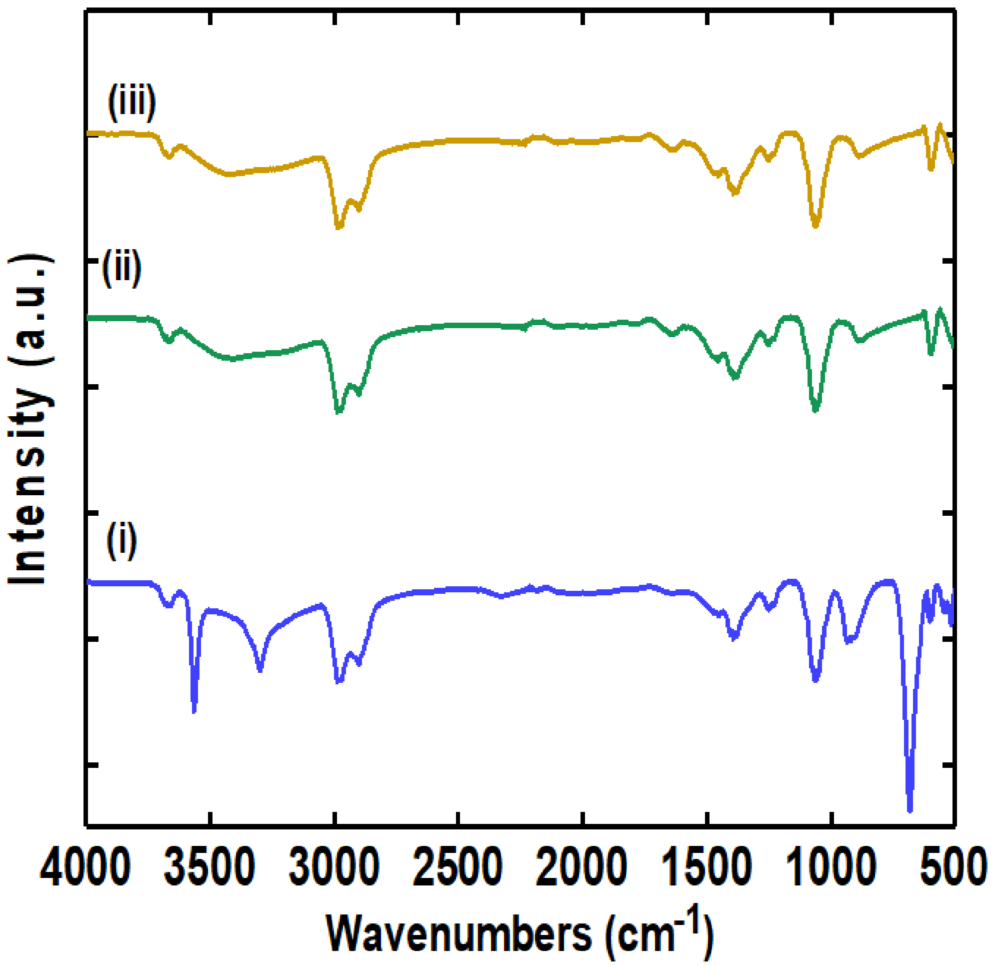

Figure S2: FTIR spectra for (i) $Cu(OH)_2$ nanofiller, (ii) CuO-200 nanofiller and (iii) CuO-500 nanofiller

Figure S2(i) shows that there are the appearance of stretching modes of O-H groups in $Cu(OH)_2$ at the peaks of 3568 cm^{-1} and 3305 cm^{-1}. The bands at 1380 and 1060 cm^{-1} indicate the bending mode of the absorbed water in as-prepared of $Cu(OH)_2$ powder [27]. While, at 933 cm^{-1} attributes to the C-O stretching vibration of metal cation, Cu^{2+} in $Cu(OH)_2$.

Figure S2 (ii) and (iii) shows that the bands at 3440 and 1628 cm^{-1} for CuO-200 and CuO-500 correspond to the O-H group of water. While the absorption bands observed at 594 cm^{-1} is referring to Cu-O stretching modes [28]. Thus, the FTIR results confirm the formation of CuO at all calcination temperatures.

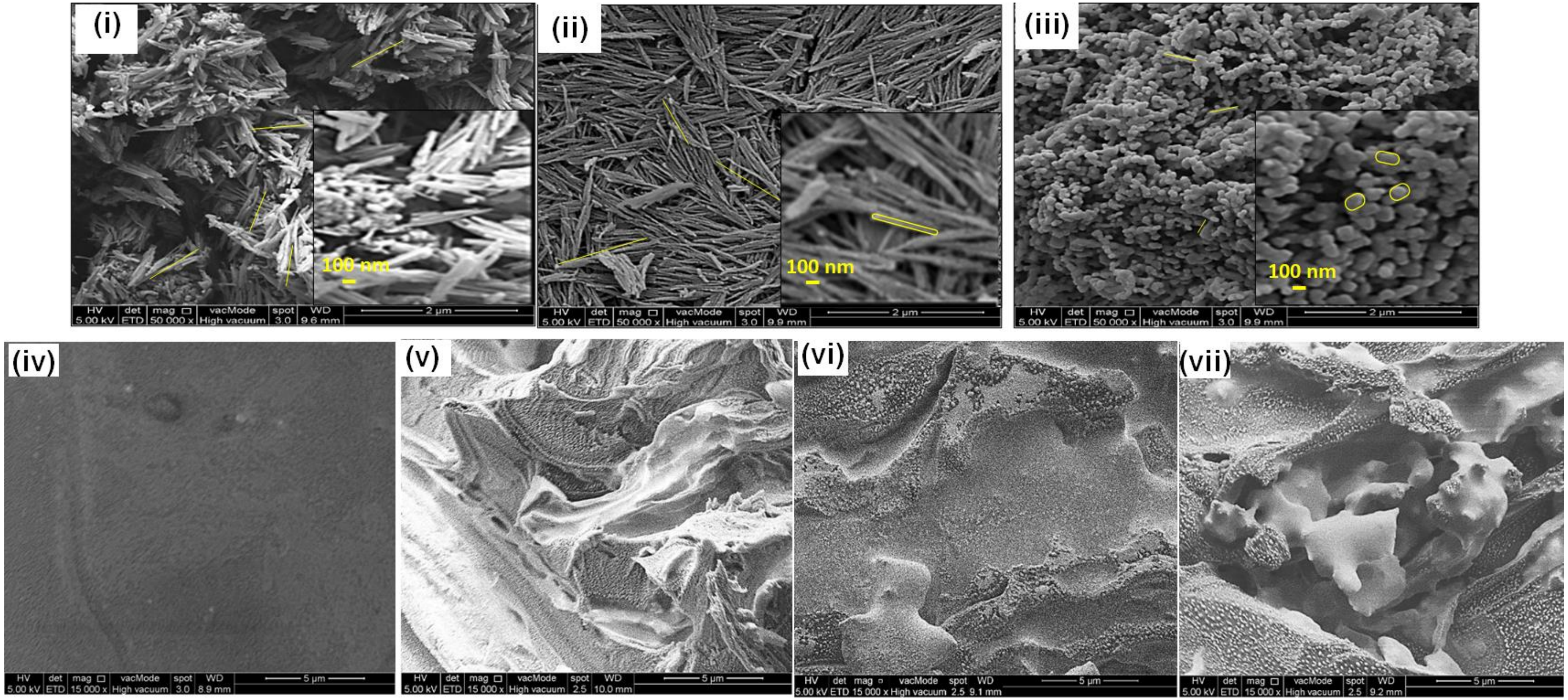

Figure S3: FESEM micrographs for (i) $Cu(OH)_2$ nanofiller, (ii) CuO-200 nanofiller, (iii) CuO-500 nanofiller, (iv) Pure P(VB-co-VA-co-VAc), (v) $TCu(OH)_2$, (vi) TCuO-200 and (vii) TCuO-500.

Table S1: Size and shape of Cu(OH) and CuO nanofiller at different calcination temperature.

**Nanofiller**	**Size of nanofiller**	**Shape of naofiller**
$Cu(OH)_2$	Average length: 0.75 µm, diameter: 45 nm	nanorod
CuO-200	Average length: 1.12 µm, diameter: 45-50 nm	nanorod
CuO-350	Range of length: 1.58 consist of small nanorods (average length: 227 nm, diameter:91 nm)	Interconnected chain of small rod
CuO-500	diameter: 182 nm	sphere
